# Proportionality of Clinical Outcome and Placental Changes to the Increasing Severity of Maternal Hypertension- An Observational Study*

**DOI:** 10.5146/tjpath.2021.01563

**Published:** 2023-01-15

**Authors:** Priyadharshini Bargunam, Parvathi Jigalur, Purushotham Reddy

**Affiliations:** Department of Pathology, Karnataka Institute of Medical Sciences, Karnataka, India

**Keywords:** Placental Infarct, Maternal Hypertension, Preeclampsia, Eclampsia, IUGR, Fetal death, Increased Syncytial knots, Hypermature Villi

## Abstract

***Objective:*** Preeclampsia and eclampsia remain the major causes of maternal and perinatal mortality and morbidity worldwide, causing 12–15% of direct maternal deaths. Although preeclampsia and related hypertensive disorders of pregnancy continue to affect 8% of all pregnancies, the incidence of preeclampsia has increased 40% in recent years. This study was carried out to analyse the different placental lesions and fetal outcome in different grades of maternal hypertension and to see if there is a linear relationship of the same.

***Material and Method:*** A total of 539 placenta specimens received at the department of Pathology from October 2017 to March 2019 were collected after obtaining informed consent. Of the 539 placentas, 87 hypertensive cases were graded and grouped according to the severity as gestational hypertension, mild preeclampsia, severe preeclampsia, eclampsia, and chronic hypertension and compared with 88 normotensive cases. The gross and microscopic findings were tabulated and analysed using the Statistical Package for the Social Sciences (SPSS) software.

***Results:*** Incidence of fetal death and growth restriction increased with increasing grade of maternal hypertension (p= 0.001). Abnormal shape of placenta (p= 0.034) and abnormal umbilical cord insertion (p= 0.028) were seen significantly more in the hypertensive group than in the normotensive group. Infarct and abnormal vasculo-syncytial membrane (p< 0.05) and abnormal villous maturation (p= 0. 039) were significantly increased in the hypertensive group than the normotensive group.

***Conclusion:*** The incidence of adverse fetal outcome and placental changes suggestive of feto-maternal malperfusions shows a proportional trend with the increasing grade of maternal hypertension.

## INTRODUCTION

Preeclampsia and eclampsia remain the major causes of maternal and perinatal mortality and morbidity worldwide, causing 12–15% of direct maternal deaths (1,2). Although preeclampsia and related hypertensive disorders of pregnancy continue to affect 8% of all pregnancies, the incidence of preeclampsia has seen a 40% increase in recent years (3). Placental maternal and fetal vascular malperfusion lesions are independently associated with increased risk for preeclampsia recurrence (4). Hence, unlike placental examination in other cases wherein it’s just an autopsy post exposure, detailed examination of the placenta and documentation of the placental lesions in maternal hypertension can provide valuable data that can prevent and predict the same in future pregnancies. This study was carried out to analyse the various placental lesions and fetal outcome in different grades of maternal hypertension and to see if there is a linear relationship of the same.

## MATERIALS and METHODS

All placenta specimens received at the department of Pathology from October 2017 to March 2019 were collected after obtaining informed consent. All the cases with maternal hypertension, irrespective of the gestational age and fetal outcome were included in this study. Unbooked cases and cases without clinical history and imaging findings were excluded. Out of the 539 cases included in this study, 87 cases had a maternal history of hypertension. These placentas were compared with 88 other normotensive placentas grossly and microscopically and the results were analysed for statistical significance. The 87 hypertensive cases were graded and grouped according to the severity as gestational hypertension, mild preeclampsia, severe preeclampsia, eclampsia, and chronic hypertension.

The collected placenta specimens were preserved in 10% formalin immediately after delivery. Intact specimen were subjected to thorough gross examination for the measurement of weight, diameter and thickness and cut open by loafing it according to Amsterdam guidelines (5). After adequate fixation over a period of 24-48 hours, representative bits were taken for microscopic examination, processed and stained with haematoxylin and eosin, and studied. Special stains were used whenever required.

Parenchyma were examined for villitis, calcification, fibrin deposition (grading used), acute or chronic infarct, abnormal maturation, villous edema, fetal vessel thrombosis, endarteritis of stem villi, crowding and the status of the vasculo-syncytial membrane. Syncytial knots were counted in 100 tertiary villi and compared with the standards. Umbilical cords were examined for signs of infection and umbilical artery thrombosis. Membranes were examined for signs of infection and amniotic fluid irritation. The results were statistically analysed using Statistical Package for the Social Sciences (SPSS) version 21.

## RESULTS

### Effects on Fetal Outcome

**Figure 1:**
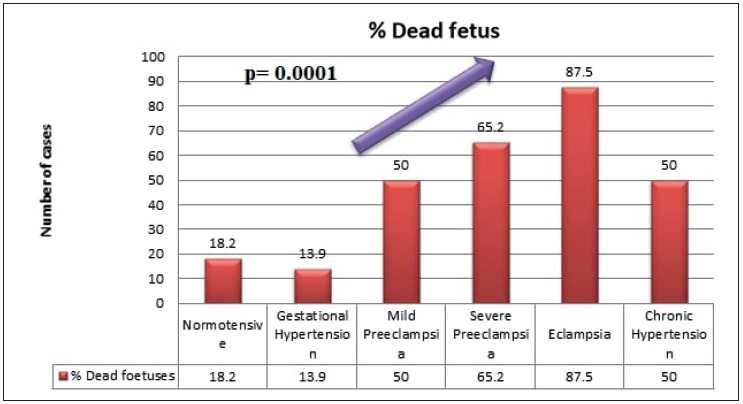
Increased percentage of fetal death with increasing grade of maternal hypertension.

**Figure 2:**
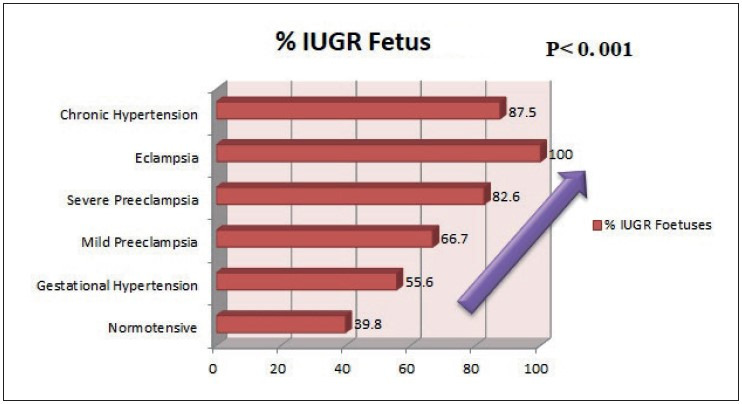
Trend of growth restriction with increasing grade of hypertension.

There was a trend observed in the study, and showed increased adverse fetal outcome such as fetal death and growth restriction with increasing grade of maternal hypertension, which was found to be statistically significant (*p= 0.001*, wherein p stands for probability value) as shown in Figure 1, Figure 2, and Table 1.

**Table 1:** Effects of maternal hypertension on placentae and fetal outcome.

	**Normotensive**	**Gestational Hypertension**	**Mild preeclampsia**	**Severe Preeclampsia**	**Eclampsia**	**Chronic Hypertension**	**p** **value**
IUGR	35 (39.8%)	20 (55.6%)	8 (66.7%)	19 (82.6%)	8 (100%)	7 (87.5%)	<0.001*
Fetal death	16 (18.2%)	5 (13.9%)	6 (50%)	15 (65.2%)	7 (87.5%)	4 (50%)	<0.001*
Increased Syncytial Knots	21 (23.9%)	14 (39.8%)	3 (25%)	5 (62.5%)	5 (62.5%)	3 (37.5%)	0.123
Infarct	4 (4.5%)	8 (22.2%)	3 (25%)	10 (43.5%)	5 (62.5%)	5 (62.5%)	<0.001*
Poor Vasculo-syncytial Membrane	15 (17%)	11 (30.4%)	1 (8.3%)	7 (30.4%)	4 (50%)	4 (50%)	0.049*
Abnormal maturation	1 (1.1%)	3 (8.34%)	2 (16.7%)	1 (4.3%)	0 (0%)	0 (0%)	0.039*
Obliterated Blood vessels	3 (3.4%)	1 (2.8%)	0 (0%)	0 (0%)	0 (0%)	1 (12.5%)	0.543
Increased Fibrin	10 (11.4%)	3 (8.34%)	0 (0%)	5 (62.5%)	1 (12.5%)	0 (0%)	0.354
Increased Calcification	9 (10.2%)	5 (5.6%)	1 (8.3%)	3 (13%)	0 (0%)	0 (0%)	0.729
Villous edema	2 (2.3%)	0 (0%)	0 (0%)	0 (0%)	0 (0%)	0 (0%)	0.849
Villitis	1 (1.1%)	3 (8.34%)	0 (0%)	1 (4.3%)	1 (12.5%)	0 (0%)	0.238
Abnormal Shape of Placenta	2 (2.3%)	6 (16.7%)	1 (8.3%)	5 (21.7%)	1 (12.5%)	1 (12.5%)	0.017*

* Statistically significant

Twelve (33.3%) cases of gestational hypertension, 6 (50%) cases of the mild preeclampsia group, 17 (73.9%) cases of severe preeclampsia, 7 (87.5%) cases of the eclampsia group, and 7 (87.5%) cases of chronic hypertension resulted in preterm delivery of the fetuses, further adding to the fetal complication (*p= 0.0001*).

### Gross Placental Changes

**Figure 3:**
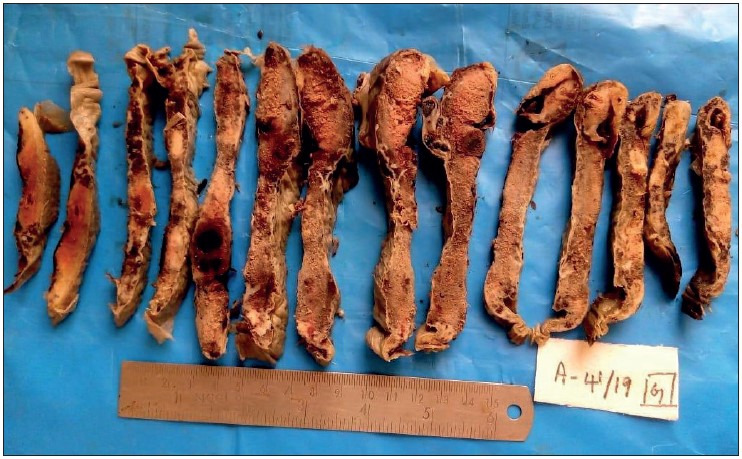
Showing placenta of 37-week-old intrauterine dead (IUD) fetus, with maternal history of severe preeclampsia, showing large areas of chronic infarct and an area of acute parenchymal thrombus, probably because of abruption. The umbilical cord was marginally inserted and microscopy revealed the thrombus with increased syncytial knots and chorioamnionitis.

**Figure 4:**
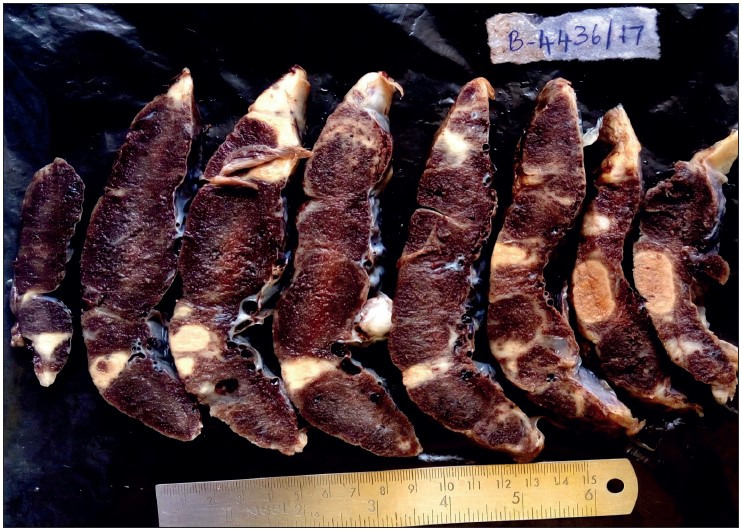
Showing placenta of 39-week IUD fetus with maternal history of gestational hypertension, showing multiple infarcts.

**Figure 5:**
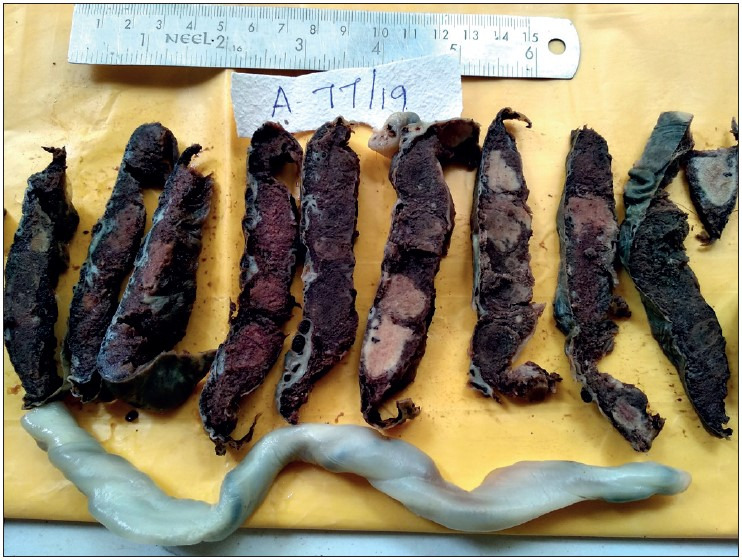
Showing placenta of a 29-week IUD fetus, with maternal history of eclampsia and Hemolysis, Elevated Liver enzymes, and Low Platelet count (HELLP) Syndrome, showing various stages of infarct- Acute and Remote. The fetus showed features of gross intra-uterine growth restriction. The umbilical cord was hypocoiled. Microscopy revealed infarct, increased syncytial knots and poor vasculo-syncytial membrane.

**Figure 6:**
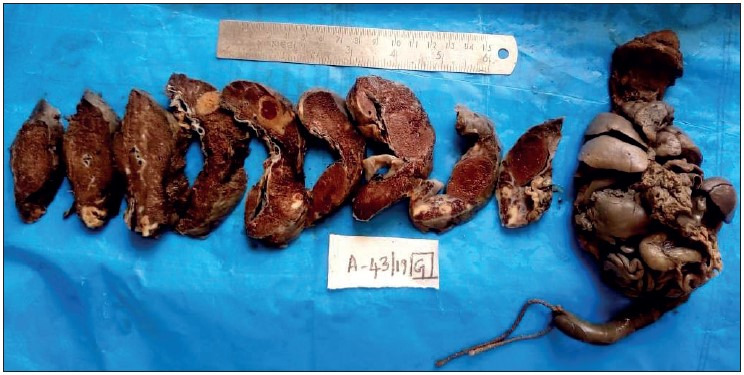
Showing cut section of placenta and en bloc 32-week dead fetus, with maternal history of eclampsia and severe anaemia. Placenta shows a parenchymal thrombus progressing to chronic infarct. Microscopy confirmed the gross placental changes. The en bloc fetus shows an absent left lung, cardiomegaly, splenomegaly, and hypoplastic liver. The cause of death here is multiple congenital anomalies and not the placental changes due to hypertension.

Abnormal shape of the placenta was seen more commonly in the hypertensive group than in the normotensive group, which was found to be statistically significant (*p= 0.034*). Abnormal shape was seen the most in gestational hypertension in 6 (37.5%) cases followed by severe preeclampsia in 5 (31.3%) cases. Abnormal umbilical cord insertion was significantly increased in the hypertensive group compared to the normotensive group (*p= 0.028*). Figure 3, Figure 4, Figure 5, Figure 6 show various gross changes in the placentas of the hypertensive group.

### Microscopic Changes

**Figure 7:**
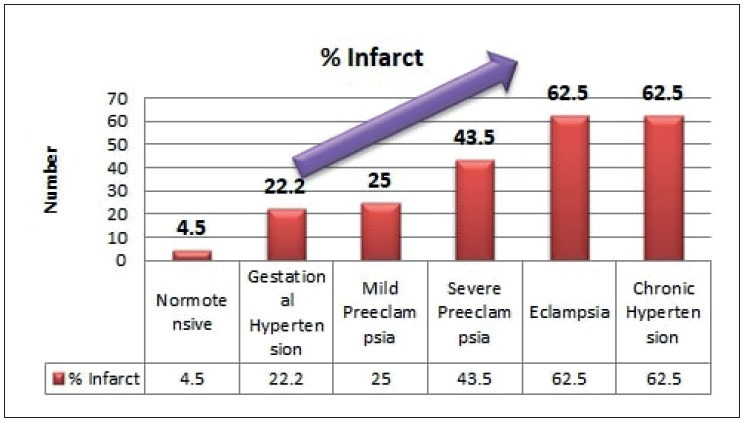
Infarct (%) in different grades of maternal hypertension.

**Figure 8:**
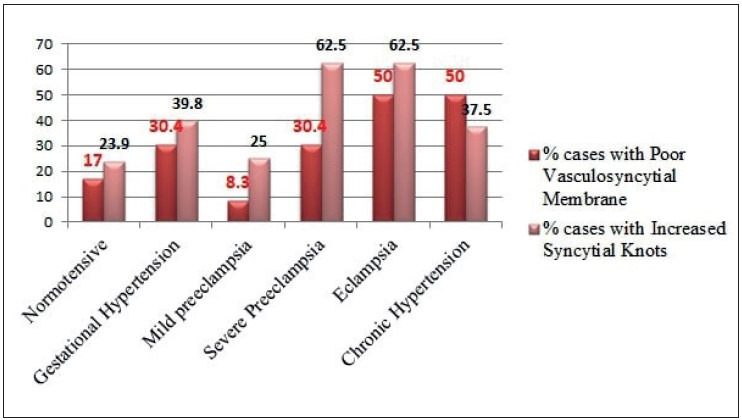
Syncytial knots and vasculo-syncytial membrane status in increasing grades of maternal hypertension.

**Figure 9:**
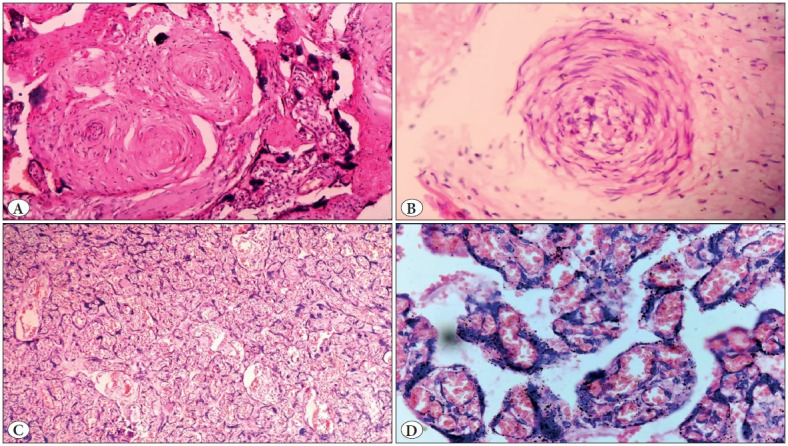
Showing microscopic images of a case of antepartum eclampsia**, A)** Obliterated and spasmodic blood vessels in a stem villus (100x magnification), **B)** Acute atherosis of blood vessels with infiltration by foamy cells (300x), **C)** Congested blood vessels and haemorrhage with increased syncytial knots (400x), **D)** Crowded and congested villi with increased syncytial knots (10x).

**Figure 10:**
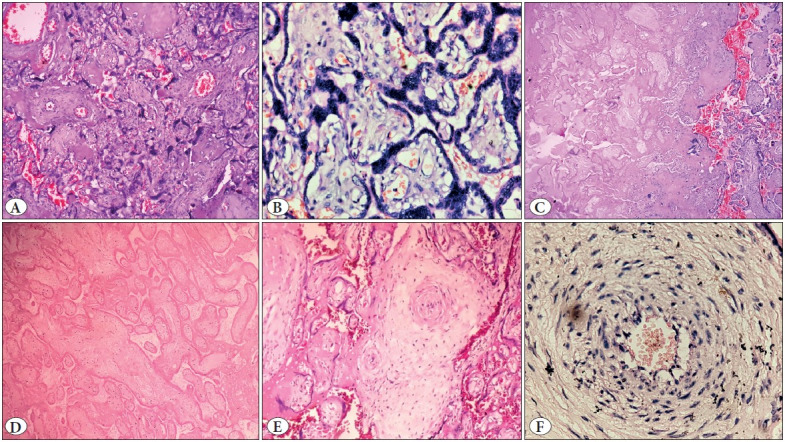
Showing microscopic images of a case of severe preeclampsia diagnosed at 26 week of gestation. **A)** Crowded villi with increased syncytial knots and perivillous fibrin (40x), **B)** Increased syncytial knots (400x), **C)** Area of infarct showing ghost villi as compared with the surrounding normal villi (10x), **D)** Ghost villi (100x), **E)** Obliterated vessels (40x), **F)** Acute atherosis with infiltration by foamy cells (400x).

Infarct and abnormal vasculo-syncytial membrane were seen increasing significantly with increasing grade of hypertension (*p< 0.05*), as shown in Figure 7, Figure 8, and Table 1. Abnormal villous maturation was increased in the hypertensive group than the normotensive group (*p= 0. 039*). Figure 9 and Figure 10 show various microscopic changes in the placentas of the hypertensive group.

## DISCUSSION

In this study, 87 placentas with a maternal history of hypertension were compared with 88 other normotensive placentas grossly and microscopically and the results were analysed. The 87 hypertensive cases were graded and grouped according to the severity as gestational hypertension, mild preeclampsia, severe preeclampsia, and eclampsia according to* *American College of Obstetricians and Gynecologists (ACOG) Guidelines and compared with chronic hypertensive and normotensive cases.

Hypertension was classified and graded as follows as per the ACOG guidelines: (6)

- Gestational Hypertension: Blood pressure >140/90 mm Hg (millimetres of mercury) presenting after 20 weeks of pregnancy without significant proteinuria.
- Preeclampsia: Blood pressure >140/90 mmHg presenting after 20 weeks of pregnancy with significant proteinuria (>30 mg/ml (milligrams/millilitre), or >300 mg/day or at least 1g/L (grams/litre) [2+] on dipstick testing).
- Severe Preeclampsia: Blood pressure >160/110 mm Hg presenting after 20 weeks of pregnancy with significant proteinuria (>30 mg/ml, or >300 mg/day or at least 1g/L [2+] on dipstick testing) with symptomatic manifestation.
- Eclampsia: Eclampsia was defined as the presence of new-onset grand mal seizures in a woman with preeclampsia. Eclampsia can occur before, during or after labour.

Chronic hypertension is high blood pressure that either precedes pregnancy, or is diagnosed within the first 20 weeks of pregnancy, or does not resolve by the 12-week postpartum check-up. Two categories of severity are recognized: mild (up to 179 mmHg systolic and 109 mmHg) and severe (≥180 systolic or 110 diastolic). Chronic hypertension complicates about 5% of all pregnancies, and prevalence rates are increasing due to delayed childbearing (7).

Although the exact pathogenesis is unknown, inadequate trophoblast invasion leading to incomplete remodelling of the uterine spiral arteries is considered to be a primary cause of placental ischemia (8). Thus the poorly perfused and hypoxic placenta is thought to synthesize and release increased amounts of vasoactive factors such as soluble fms-like tyrosine kinase-1 (sFlt-1), cytokines, and possibly the angiotensin II (ANG II) type 1 receptor autoantibodies (AT1-AA) and these are thought to induce widespread activation/dysfunction of the maternal endothelium in the vessels of the kidney and other organs that ultimately results in hypertension (8–10). It is postulated that the ischemic placenta contributes to endothelial cell dysfunction in the maternal vasculature by inducing an alteration in the balance of circulating levels of angiogenic/antiangiogenic factors such as vascular endothelial growth factor (VEGF), placental growth factor (PlGF), and sFlt-1 (11,12), though sFlt-1 levels play a key role in initiating the symptoms of preeclampsia (11,13).

Classic pathologic findings in the placenta associated with hypertensive disease during pregnancy include lesions of uteroplacental malperfusion such as decidual vasculopathy, placental infarctions, ischemic changes (such as increased syncytial knots, villous agglutination, accelerated villous maturation, and villous hypovascularity), increased perivillous fibrin, and chronic deciduitis (14–16).

A recent meta-analysis done to calculate the recurrence rate of hypertensive disorder of pregnancy concluded that the recurrence rate in the subsequent pregnancy was 20.7% and the recurrence rate was 13.8% specifically for preeclampsia (17), reiterating the need for placental examination in all cases of maternal hypertension.

There was a trend observed in this study, and showed increased adverse fetal outcome such as fetal death and growth restriction with increasing grade of maternal hypertension. This is in comparison with the study done by Navbir. (18).

The placental weight is a surrogate for placental function (19), and the feto-placental weight ratio has been suggested as a possible indicator of adequacy of placental reserve capacity in fetal growth restriction (FGR) (20). Kher and Zawar and several other studies elsewhere (21,22) have reported a significant reduction in the feto-placental weight ratio. Fox and Sebire (23) explained that this decreased feto-placental weight ratio is because of a compensatory hypertrophy of the placenta under the influence of the unfavourable maternal environment. In our study, the feto-placental weight ratio was not calculated because we couldn’t weigh placentas without fixation errors. The Amsterdam Consensus Placental Sampling guidelines state that the placenta should be weighed after trimming the extra placental membranes and umbilical cord, and notation of whether the placenta was fresh or fixed when measured and should be compared with contemporary placental weight standards derived from the respective local or similar population (5). Fixation of the placenta will affect its weight, with an increase of 3% to 6% (24). Few samples were received from remote parts of Karnataka, which had been preserved in formalin for days before they reached our department. That caused considerable variation in the weight, causing lack of uniformity, which is a drawback of this study.

Preterm delivery is a leading cause of perinatal morbidity and mortality and a major cost burden to the health care system. In our study, 12 (33.3%) cases of gestational hypertension, 6 (50%) cases of mild preeclampsia group, 17 (73.9%) cases of severe preeclampsia, 7 (87.5%) cases of eclampsia group, and 7 (87.5%) cases of chronic hypertension resulted in preterm delivery of the fetuses, further adding to the fetal complication (*p= 0.0001*).

Abnormal shape of placenta was seen more in the hypertensive group than in the normotensive group, which was found to be statistically significant (*p= 0.034*). Abnormal shape was seen most in gestational hypertension in 6 (37.5%) cases followed by severe preeclampsia in 5 (31.3%) cases. Abnormal umbilical cord insertion was significantly increased in the hypertensive group than in the normotensive group (*p= 0.028*).

Infarct and abnormal vasculo-syncytial membrane were seen to increase significantly with increasing grade of hypertension (*p< 0.05*), as shown in Figure 7, Figure 8 and Table 1. Ezeigwe et al. concluded that in preeclampsia/ eclampsia the degree of placental involvement by infarction was inversely proportional to fetal birth weight (25).

In our study, abnormal villous maturation was seen to increase more in the hypertensive group than in the normotensive group (*p= 0. 039*). Ruiz-Quiñonez et al. (26) concluded that the Placenta Maturity Index {PMI= number of vasculo-syncytial membranes (VSM) in 1 mm2 divided by VSM thickness (mm)} was increased in preeclampsia, but not in gestational hypertension. Placental hypermaturity was also associated with the diagnosis of Small for Gestational Age (SGA) in newborns.

A retrospective case–control study conducted by Devisme et al. (27) found that infarcts (65.9% versus 13.2%; *p< 0.001*) and placental abruption (*p< 0.001*) were most frequent among women with preeclampsia. Increased syncytial knots (90% versus 9%; *p< 0.001*), infarcts, basal decidual vasculopathy (51% vs. 8%; *p< 0.001*), hypermature villi (72% vs. 16%; *p< 0.001*) were significantly associated with pre-eclampsia, which is comparable with our study.

A meta- analysis done by Falco et al. (28) concluded that both placental villous and vascular histopathological lesions is higher, by a factor of four to seven-fold, in preeclampsia compared to normal pregnancies, though the lesions are not specific for preeclampsia. Gibbins et al. (29) performed a population-based cohort study of all stillbirths and a sample of live births from 2006 to 2008 in five catchment areas and compared placental pathology between stillbirths and those with and without Preeclampsia/Gestational Hypertension (PE/ GH) and found that parenchymal infarctions are more common in PE/GH preterm stillbirths with significant overlap in lesions found in stillbirths and PE/GH.

Stanek et al. (30) studied different grades of hypertension and its effects on the placenta and concluded that the preeclamptic groups showed the highest rates of decidual arteriolopathy, which is comparable with our study. Salafia et al. (31) concluded that placental infarction was associated with increasing levels of proteinuria. Kovo M., et al. (21) compared pregnancy outcome and placental pathology in pregnancies complicated by fetal growth restriction (FGR) with and without preeclampsia and found higher rate of maternal placental vascular lesions in hypertensive-FGR compared with normotensive-FGR (82% versus 57.7%, *p<.001*).

## CONCLUSION

The incidence of adverse fetal outcome and placental changes suggestive of feto-maternal malperfusion shows a linear trend with the grade of maternal hypertension.

## Conflict of Interest

All authors declare that they have no conflict of interest.
